# C-type natriuretic peptide ameliorates pulmonary fibrosis by acting on lung fibroblasts in mice

**DOI:** 10.1186/s12931-016-0335-6

**Published:** 2016-02-19

**Authors:** Toru Kimura, Takashi Nojiri, Jun Hino, Hiroshi Hosoda, Koichi Miura, Yasushi Shintani, Masayoshi Inoue, Masahiro Zenitani, Hiroyuki Takabatake, Mikiya Miyazato, Meinoshin Okumura, Kenji Kangawa

**Affiliations:** Department of Biochemistry, National Cerebral and Cardiovascular Center Research Institute, 5-7-1, Fujishirodai, Suita-city, Osaka, 565-8565 Japan; Department of General Thoracic Surgery, Osaka University Graduate School of Medicine, Suita-City, Osaka, Japan; Department of Regenerative Medicine and Tissue Engineering, National Cerebral and Cardiovascular Center, Suita-City, Osaka, Japan; Department of General Thoracic Surgery, Kyoto Prefectural University of Medicine, Kyoto-City, Kyoto, Japan

**Keywords:** C-type natriuretic peptide, Lung fibroblast, Pulmonary fibrosis, Bleomycin, Transforming growth factor-β

## Abstract

**Background:**

Pulmonary fibrosis has high rates of mortality and morbidity; however, no effective pharmacological therapy has been established. C-type natriuretic peptide (CNP), a member of the natriuretic peptide family, selectively binds to the transmembrane guanylyl cyclase (GC)-B receptor and exerts anti-inflammatory and anti-fibrotic effects in various organs through vascular endothelial cells and fibroblasts that have a cell-surface GC-B receptor. Given the pathophysiological importance of fibroblast activation in pulmonary fibrosis, we hypothesized that the anti-fibrotic and anti-inflammatory effects of exogenous CNP against bleomycin (BLM)-induced pulmonary fibrosis were exerted in part by the effect of CNP on pulmonary fibroblasts.

**Methods:**

C57BL/6 mice were divided into two groups, CNP-treated (2.5 μg/kg/min) and vehicle, to evaluate BLM-induced (1 mg/kg) pulmonary fibrosis and inflammation. A periostin-CNP transgenic mouse model exhibiting CNP overexpression in fibroblasts was generated and examined for the anti-inflammatory and anti-fibrotic effects of CNP via fibroblasts in vivo. Additionally, we assessed CNP attenuation of TGF-β-induced differentiation into myofibroblasts by using immortalized human lung fibroblasts stably expressing GC-B receptors. Furthermore, to investigate whether CNP acts on human lung fibroblasts in a clinical setting, we obtained primary-cultured fibroblasts from surgically resected lungs of patients with lung cancer and analyzed levels of GC-B mRNA transcription.

**Results:**

CNP reduced mRNA levels of the profibrotic cytokines interleukin (IL)-1β and IL-6, as well as collagen deposition and the fibrotic area in lungs of mice with bleomycin-induced pulmonary fibrosis. Furthermore, similar CNP effects were observed in transgenic mice exhibiting fibroblast-specific CNP overexpression. In cultured-lung fibroblasts, CNP treatment attenuated TGF-β–induced phosphorylation of Smad2 and increased mRNA and protein expression of α-smooth muscle actin and SM22α, indicating that CNP suppresses fibroblast differentiation into myofibroblasts. Furthermore, human lung fibroblasts from patients with or without interstitial lung disease substantially expressed GC-B receptor mRNA.

**Conclusions:**

These data suggest that CNP ameliorates bleomycin-induced pulmonary fibrosis by suppressing TGF-β signaling and myofibroblastic differentiation in lung fibroblasts. Therefore, we propose consideration of CNP for clinical application to pulmonary fibrosis treatment.

## Background

Idiopathic pulmonary fibrosis (IPF) is a chronic disease characterized by progressive scarring of the lung parenchyma [1]. Recent guidelines for IPF show that no effective pharmacological therapy has been established and that the 5-year survival rate of IPF is less than 50 % [1]. More recently, pirfenidone and nintedanib were approved for the treatment of IPF due to evidence of their efficacy in slowing functional decline and disease progression [2]. Although the pathologic processes that cause disease progression are not fully understood, IPF is characterized by a microscopic pattern of usual interstitial pneumonia, which includes excessive collagen deposition, honeycombing, and the presence of fibroblastic foci [3]. Fibroblasts, which can be activated to differentiate into highly secretory and contractile smooth muscle-like cells termed myofibroblasts, are mesenchymal cells that serve a critical role in both normal and fibrotic repair processes [4]. Fibroblastic foci are areas of myofibroblast proliferation thought to be the main site of abnormal collagen deposition [5]. Transforming growth factor (TGF)-β is a key mediator of fibrosis in many tissues including lung. In pulmonary fibrosis, TGF-β stimulates fibroblast to myofibroblast transformation and promotes collagen deposition of fibroblasts [6–8]. Thus, the lung fibroblasts and myofibroblasts within fibroblastic foci represent an attractive target for the treatment of IPF [7].

We originally isolated C-type natriuretic peptide (CNP) from porcine brain as the third member of the natriuretic peptide family [9], the other members being atrial and brain natriuretic peptide (ANP and BNP, respectively). CNP selectively binds to the transmembrane guanylyl cyclase (GC)-B receptor and subsequently leads to a large increase in intracellular cyclic guanosine monophosphate (cGMP) [10]. CNP is expressed in a wide variety of tissues, such as vascular endothelium, heart, bone, and adrenal and reproductive glands [11–15]. CNP plays a role in the local regulation of vascular tone and remodeling, and has been shown to have mainly cardioprotective effects, such as an anti-inflammatory effect, in a rat model of myocarditis [16] and anti-hypertrophic and anti-fibrotic effects in a rat model of myocardial infarction [17]. Recently, CNP was shown to have protective effects against inflammatory and fibrotic reactions in articular cartilage [18, 19], kidney [20, 21], and skin [22]. Murakami et al. [23] reported that CNP modulated the BLM-induced inflammatory reaction by reducing the production of inflammatory cell-attracting chemokines, but the precise mechanisms were unclear. We recently revealed that exogenous CNP attenuates lipopolysaccharide (LPS)-induced acute lung injury in mice [24]. CNP is synthesized by cardiac fibroblasts as well as vascular endothelial cells in neonatal rats, and CNP plays a role as an autocrine regulator against excessive cardiac fibrosis and secretion of inflammatory cytokines [25, 26]. Thus, fibroblasts, which exist in various organs and are involved in fibrosis, are likely target cells of CNP.

Bleomycin (BLM)-administered mice are widely used as a model of IPF [27]. The administration of BLM causes epithelial injury, followed by neutrophil-dominant and lymphocyte-dominant inflammation that leads to fibrosis [28]. In the inflammatory phase, the expression levels of various chemokines, cytokines, and growth factors are elevated, and these mediators exert their profibrotic activities through the activation and proliferation of fibroblasts [28]. Considering the pathophysiological importance of fibroblast activation in pulmonary fibrosis [4, 28, 29] and the above-mentioned reports about CNP and cardiac fibroblasts [25, 26], we hypothesized that the anti-fibrotic and anti-inflammatory effects of exogenous CNP against BLM-induced pulmonary fibrosis were exerted at least in part by the effect of CNP on pulmonary fibroblasts.

Here we showed that CNP attenuated the inflammatory reaction and fibrotic changes induced by BLM administration in murine lung, and that BLM-induced pulmonary inflammation and fibrosis were attenuated in transgenic mice whose fibroblasts overexpressed CNP. Additionally, we demonstrated the suppressive effect of CNP in vitro on the activation of human lung fibroblasts by TGF-β. These results suggest that the target of the anti-fibrotic effect of CNP is lung fibroblasts. Finally, we demonstrated that the mRNA of GC-B was expressed in primary-cultured human lung fibroblasts from patients with or without interstitial lung disease (ILD). These insights suggest the possibility of CNP as a new therapeutic agent for patients with ILD including IPF.

## Methods

### Animal studies

C57BL/6N mice (male, 6 weeks old, weighing 18–20 g each) were purchased from Japan SLC (Shizuoka, Japan). Animals were maintained at a controlled temperature of 24 °C ± 1 °C with a 12:12 h light–dark cycle (light cycle, 07:00–19:00), and were fed a standard diet. Water was freely available. All experimental protocols described herein were approved by the Animal Care Ethics Committee of the National Cerebral and Cardiovascular Center Research Institute, Japan.

### Generation of periostin-CNP transgenic mice

The periostin–CNP transgenic construct, which drives CNP expression under the control of the periostin promoter, was generated by using the Red/ET Counter Selection bacterial artificial chromosome (BAC) modification Kit (Gene Bridges, Heidelberg, Germany). A human BAC clone RP11-46 M10 containing the CNP gene and a mouse BAC clone RP23-144B14 containing the periostin gene (Thermo Fisher Scientific/Invitrogen, MA, USA) were used to construct the transgene [30]. The periostin-CNP transgenic construct was purified and microinjected into the pronucleus of C57BL/6 J mouse embryos by use of standard techniques. Transgenic F1 mice were identified by Southern blot analysis of tail DNA, and were then mated with C57BL/6 J mice to produce a large number of periostin-CNP transgenic mice. The probe for the Southern blot analysis was prepared by polymerase chain reaction (PCR) with the following primers: intron forward (5′-GGCTGTCTCCTCCGAGATG-3′), and SV40 reverse (5′-TGAGTTTGGACAAACCACAACTAGA-3′). The heterozygosity of the transgenic mice was maintained.

### Analysis of transgene expression

Total RNA was prepared from mouse tissues as follows. PCR amplification was performed with AmpliTaq Gold polymerase (Life Technologies, Inc.) according to the manufacturer’s instructions. To distinguish transgene expression from endogenous CNP expression, the human CNP exon sequence and the untranslated sequence of the transgene were used as the forward primer and reverse primer, respectively (forward: 5′-AAGAAGGGCTTGTCCAAGGG-3′; reverse: 5′-GTTTCAGGTTCAGGGGGAGG-3′). Internal control RT-PCR was performed with the 36B4 gene.

### Chemicals and reagents

BLM, purchased from Nippon Kayaku Co. (Tokyo, Japan), was dissolved in normal saline and adjusted to the appropriate concentrations as described below. CNP, purchased from Peptide Institute, Inc. (Osaka, Japan), was dissolved in 5 % w/v glucose solution at a concentration of 20 mg/mL.

### BLM administration and CNP treatment

BLM (1 mg/kg) in 80 μl of saline was administered via oropharyngeal aspiration as previously described [31]. CNP (2.5 μg/kg/min) or vehicle was subcutaneously (not intravenously) infused by using an osmotic mini-pump (Alzet Model 1003D, Duret Corporation, Cupertino, CA, USA) and the pumps were implanted 24 h before BLM administration as previously described [24]; the infusion continued until the mice were euthanized. This protocol resulted in the creation of three groups: normal control mice, BLM-administered mice treated with CNP, and BLM-administered mice treated with vehicle (*n* = 18 in each group).

### Bleomycin administration

The mice were anesthetized with 3 % isoflurane delivered in a box, and BLM (1 mg/kg) in 80 μL of saline or vehicle alone was administered via oropharyngeal aspiration with a micropipette (day 0). Oropharyngeal aspiration was performed as described by de Vooght et al. [31] Mice were fixed on a surgery board, the tongue was pulled out with the use of forceps, and the liquid was placed onto the distal part of the oropharyngeal airway while the nose was gently closed. Mice were killed on day 14. A subgroup of the mice was assessed by measuring cell counts in bronchoalveolar lavage (BAL) fluid as described below, while the remainder were euthanized for histological, gene expression, and hydroxyproline analysis of the lung. The left lung was fixed by intratracheal instillation of 4 % paraformaldehyde for 24 h and subsequently embedded in paraffin. Paraffin sections were stained with hematoxylin-eosin and Masson trichrome.

### BAL fluid analysis

BAL fluid was assessed as previously described [32, 33]. In an open-chest procedure, mouse trachea was cannulated (20-gauge intravenous catheter) and 1 mL of PBS was infused intratracheally and withdrawn. This lavage technique was repeated two additional times with the same 1.0 mL solution. BAL fluids were centrifuged at 300 × *g* for 10 min at 4 °C. The cell pellet was suspended in 0.5 mL of PBS, and slides were prepared by cytocentrifugation (Cytospin 4, Thermo Shandon, Pittsburgh, PA, USA) and stained with Diff-Quick (Dade Behring, Dudingen, Switzerland). Cell counts of total cells, macrophages, and neutrophils in the BAL fluid were determined by using morphological criteria under a light microscope; ≥1000 cells/slide were evaluated.

### Quantitative evaluation of lung fibrosis

Lung sections were stained with Masson trichrome, and then each slide was scanned completely in a zigzag fashion and the percentage of fibrotic area in the whole lung field was assessed. Brightfield images of Masson trichrome-stained slides were made on an FSX100 system (Olympus, Tokyo, Japan) and the fibrotic area expressed as a percentage of the whole lung field was analyzed by using CellSens Dimension software version 1.6 (Olympus).

### Hydroxyproline assay

Total lung collagen was determined by analysis of hydroxyproline on day 14 after bleomycin infusion as previously described [34]. In brief, the left lung was homogenized in 6 N hydrochloric acid and hydrolyzed at 110 °C overnight. Citrate/acetate buffer (pH 6.5) and chloramine T solution were added at room temperature for 20 min. The mixture was then incubated with Ehrlich’s solution at 65 °C for 20 min. After the samples were cooled to room temperature, the absorbance of each sample at 550 nm was measured. Hydroxyproline content was calculated from a standard curve of L-hydroxyproline (Wako Pure Chemical Industries, Ltd., Osaka, Japan).

### Gene expression analysis

Total RNA from lung and other tissues was homogenized in guanidium-phenol-chloroform and isolated by using an RNeasy mini kit (Qiagen, Hilden, Germany). Total RNA from cells in vitro was isolated by using a QIAshredder (Qiagen) and an RNeasy mini kit. The RNA was then reverse-transcribed into cDNA by using a SuperScript II Reverse Transcriptase kit (Invitrogen, Carlsbad, CA, USA) or a QuantiTect Reverse Transcription kit (Qiagen). Quantitative PCR assays were conducted in a 96-well plate with the use of SYBR Premix Ex Taq (Takara, Siga, Japan) and a Light Cycler 480 System II (Roche Applied Science, Indianapolis, IN, USA). Sequences of the primers were listed in Table 1. The PCR settings were as follows: initial denaturation for 30s at 95 °C followed by 38 cycles of 5 s at 95 °C, 20 s at 57 °C (IL-6), or 5 s at 95 °C, 10 s at 56 °C, 15 s at 72 °C (IL-1β), or 5 s at 95 °C, 20 s at 60 °C (bFGF, TGF-β, and 36B4), or 5 s at 95 °C, 20 s at 58 °C (collagen 1A, TIMP1, and SM22α), or 5 s at 95 °C, 20 s at 59 °C (α-SMA and GC-B). Melting curve analysis was conducted with the temperature increasing from 72 °C to 98 °C. Quantification of gene expression was calculated relative to the housekeeping gene 36B4.

**Table 1:** Sequence of primers used in the study

Genes	Forward (5′-3′)	Reverse (5′-3′)
IL-1β	AGCACCTTCTTTCCCTTCATCTTTG	GAGGTGGAGAGCTTTCAGTTCATAT
IL-6	CCAGTTGCCTTCTTGGGACTGATG	GTAATTAAGCCTCCGACTTGTGAAG
bFGF	GCTCTACTGCAAGAACGGCGGCTTC	ACACACTTAGAAGCCAGCAGCCGTC
Collagen 1A	AGTAACGTCGTGCCTAGCAACATGC	GAATACTGAGCAGCAAAGTTCCCAG
TIMP-1	ATCATCGAGACCACCTTATACCAGC	TGCAGGCAGTGATGTGCAAATTTCC
TGF-β	CAACTACTGCTTCAGCTCCACAGAG	CAAGGACCTTGCTGTACTGTGTGTC
α-SMA	TGCTGGCATCCATGAAACCA	GTTTGCTGATCCACATCTGC
SM22α	CCCTCCATGGTCTTTAAGCAGATGGA	TCATAAACCAGTTGGGATCTCCACGG
GC-B	GTCGCTGCGGGGATCCAGTTACG	ATGTTGGGAGGGTCTATGCAGGC
36B4	TCATTGTGGGAGCAGACAATGTGGG	AGGTCCTCCTTGGTGAACACAAAGC

### Western blot analysis

Cultured cells were lysed in NP-40 buffer (1 % Nonidet P-40, 20 mM Tris–HCl (pH 7.4), 150 mM NaCl, 5 mM EDTA) supplemented with protease and phosphatase inhibitor cocktails (Nacalai Tesque, Inc., Kyoto, Japan). The sample was centrifuged at 300 × *g* at 4 °C for 15 min, and the supernatant was collected. The concentration of total protein was determined by using the Pierce 660 nm Protein Assay Reagent (Thermo Fisher Scientific, Waltham, MA, USA). The proteins were separated by 4 to 15 % SDS-PAGE (Bio-Rad, Hercules, CA, USA) and transferred to a polyvinylidene fluoride membrane (Millipore, Billerica, MA, USA). The membrane was incubated in polyvinylidene blocking reagent (Toyobo, Tokyo, Japan) at room temperature for 30 min, and then incubated at 4 °C overnight with the appropriate primary antibody diluted in Can Get Signal Solution 1 (Toyobo). The primary antibody was detected by a horseradish peroxidase-conjugated secondary antibody diluted in Tris-buffered saline (pH 7.4) containing 0.1 % Tween 20 and visualized with Luminata Forte Western HRP substrate (Millipore, Billerica, MA, USA). An image of the membrane was obtained by using a LAS-4000 mini luminescent image analyzer (Fujifilm, Tokyo, Japan), and band intensities were quantitated by using Multi Gauge software (version 3.11, Fujifilm). Protein levels were normalized to the level of GAPDH and expressed as the ratio of the level in control mice. The primary antibodies used for the analysis were as follows: anti-alpha smooth muscle actin (α-SMA) antibody (ab5694, Abcam, Cambridge, UK), anti-SM22α antibody (ab14106, Abcam), anti-Fibronectin antibody (ab2413, Abcam), GAPDH Rabbit mAb (#3683, Cell Signaling Technology, Beverly, MA, USA), Phospho-Smad2 antibody (#3101, Cell Signaling Technology), and Smad2/3 antibody (#3102, Cell Signaling Technology).

### Specimens of human lung tissue

From the lung cancer resection specimens of 8 lung cancer patients, we harvested grossly normal-appearing lung tissues that were ≥5 cm away from the tumor. Of these 8 patients, 5 had ILD including IPF.

### Isolation and primary culture of lung fibroblasts

The tissues were placed in DMEM supplemented with antibiotics for immediate transportation on ice to the laboratory. Tissues were minced into small pieces and digested for 1 h at 37 °C in HBSS (Life Technologies, Carlbad, CA, USA) containing 2 mg/mL collagenase A (10103586001; Roche Diagnostics, Mannheim, Germany). The cell suspension was filtered with a stainless steel wire mesh (hole size, 500 μm) and then a 100-μm cell strainer (BD Biosciences, San Jose, CA, USA). Cells in DMEM containing 10 % FCS were plated on 100-mm tissue-culture plates as previously described [35]. The primary cells used in the experiments were not cultured beyond 3 passages, except fibroblasts used in the in vitro studies described below which were obtained from one of the patients without ILD.

### Immortalization of human lung fibroblasts by using lentivirus that expressed human telomerase

To facilitate the in vitro studies of human normal lung fibroblasts (LFs), primary cultured LFs were immortalized with human telomerase (hTERT)-expressing lentivirus as previously described [36]. An early passage culture of LFs was infected with hTERT-expressing lentivirus (kindly provided from KAN Research Institute Inc., Kobe, Japan). After infection, cells were selected with neomycin. The resultant cell line is referred to as LF^hTERT^.

### Plasmids, transfection, and infection

The plasmid used for preparing retrovirus vector expressing GC-B-Flag (pCX4 puro GC-B-FLAG) was constructed by PCR amplification of the human GC-B gene (obtained from Promega, Madison, WI, USA) with the forward primer (GCB NotI F2), 5′- AAAGCGGCCGCACCATGGCGCTGCCATCACTTCTGCTGTTG-3′, and the reverse primer (GCB Flag R NotI), 5′- TTTGCGGCCGCTCACTTGTCATCGTCGTCCTTGTAGTCCAGGAGTCCAGGAGGTCCTTTCCGCTCTC −3′, and then introducing the amplified GC-B-Flag fragment into the *Not*I site of pCX4 puro [34]. The vector was transfected into BOSC 23 cells with pE-eco and pGp (Takara Bio, Shiga, Japan) by using FuGENE6 (Promega). After transfection, culture supernatants were collected, filtered, and used for infection. Retroviral infection of LF^hTERT^ was performed by using the retrovirus expressing GC-B-FLAG and Ecotropic Receptor Booster (Takara Bio) according to the manufacturer’s directions. After infection, cells were selected with puromycin. The resultant cell line is referred to as LF^hTERT^/GC-B.

### Assay for cGMP of cells

Cells were serum-starved for 12 h before the cGMP assay and then incubated at 37 °C with 5 % CO_2_ in DMEM containing 0.5 mM IBMX (3-isobutyl-1-methylxanthine) (Wako) and 1 × 10^−9^ to 1 × 10^−6^ M CNP or vehicle (5 % glucose) for 15 min. The reaction was terminated by addition of 400 μL of 70 % ethanol containing 0.1 M hydrochloric acid, and the cGMP concentrations were measured with a cyclic GMP radio immunoassay kit (Yamasa, Tokyo, Japan).

### Extracellular matrix–remodelling assay

Fibroblast contractility was assessed by measuring changes in the surface area of type I collagen gels mediated by fibroblasts as previously described [37]. Serum-free DMEM was used in the assay to exclude the modulation of growth factors contained in serum [37]. The gels comprised type I collagen (Gibco, A10483-01; 0.75 mg/mL) and the cell suspension (5 × 10^5^ cells/mL) in HEPES-buffered DMEM (pH 7.4). Once the gels were set, cells were maintained in serum-free DMEM. TGF-β was added to the medium at a final concentration of 1 ng/mL. CNP was added to the medium at a final concentration of 1 μM every 24 h. After the fibroblasts were cultured in the gels for 3 days, the gels were released from the plate and the diameter of the gel was measured by using an image scanner connected to a computer running the public domain NIH image analyzing software (ImageJ version 1.48; available from http://imagej.nih.gov/ij). The percentage of contraction was calculated by using the formula 100 × (well diameter – gel diameter)/well diameter.

### Ethics

Written informed consent was obtained from all patients in this study, in accordance with ethics committee requirements from the institutes and the Declaration of Helsinki. This study protocol was approved by Institutional Review Board (IRB) of Osaka University Graduate School of Medicine.

### Statistical analysis

Data were analyzed with StatView for Windows (SAS Institute Inc., Cary, NC, USA), and are expressed as means ± SEM. Between-group comparisons were performed by using the Mann–Whitney *U* test or the unpaired Student’s *t*-test. For multiple-group comparisons, one-way ANOVA, followed by the post-hoc Fisher’s least significant difference test, was used. *P* < 0.05 was considered to be significant.

## Results

### CNP ameliorated BLM-induced lung fibrosis and inflammation in mice

**Fig. 1:**
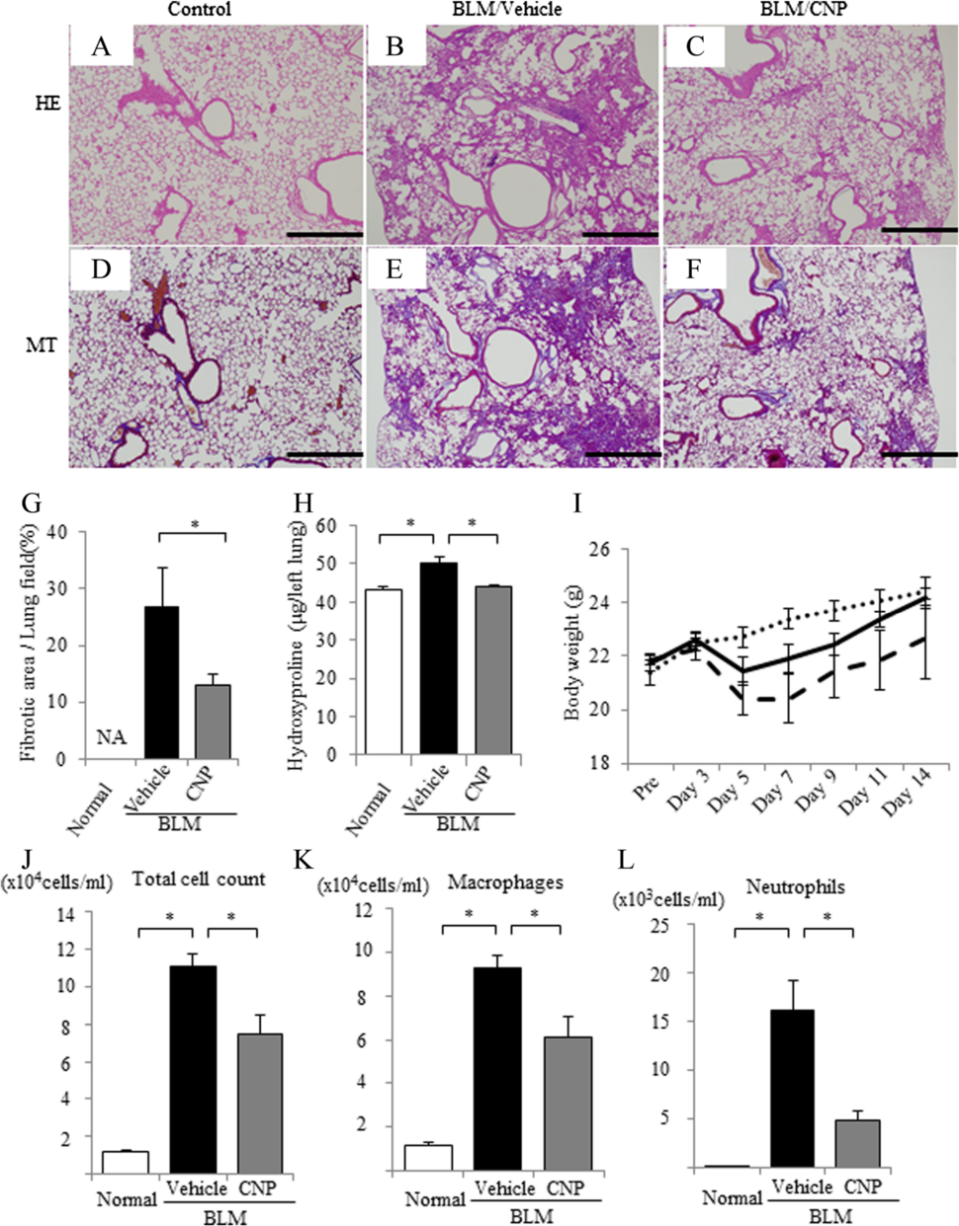
Continuous infusion of C-type natriuretic peptide (CNP) attenuated bleomycin (BLM)-induced pulmonary fibrosis in mice. BLM (1 mg/kg) was administered intratracheally to C57BL/6 mice on day 0, and the samples were removed on day 14. CNP (2.5 μg/kg/min) or vehicle (5 % w/v glucose solution) was subcutaneously infused throughout the experiment with the use of an osmotic mini-pump. Representative micrographs of lung tissue stained with hematoxylin-eosin (HE; **a–c**) and with Masson trichrome (MT; **d–f**)); normal control mice (**a**, **d**), BLM-instilled mice treated with vehicle alone (**b**, **e**) and BLM-instilled mice treated with CNP (**c**, **f**). Scale bar: 500 μm. **g** Fibrotic area was calculated by using image analyzing software and expressed as a percentage of the whole lung field. Values represent means ± SEM (*n* = 4 mice per group). *P < 0.05. NA, not assessed because of no fibrotic area in normal lungs. **h** Total collagen deposition was assessed by analyzing hydroxyproline concentrations in mouse lung tissue. Values represent means ± SEM (*n* = 6 mice per group). *P < 0.05. **i** The body weight changes of mice after BLM administration are shown for normal control mice (dotted line), BLM-instilled mice treated with vehicle alone (dashed line), and BLM-instilled mice treated with CNP (solid line). Values represent means ± SEM (*n* = 6 mice per group). **j**–**l** The numbers of total cells (**j**), macrophages (**k**), and neutrophils (**l**) in bronchoalveolar lavage (BAL) fluid on day 14 after BLM administration are shown. Values represent means ± SEM (*n* = 4 mice per group). *P < 0.05

First, we examined the in vivo anti-inflammatory and anti-fibrotic effects of CNP by using a BLM-induced lung fibrosis model in C57BL/6 mice. The histological findings confirmed that BLM administration induced lung parenchymal fibrotic lesions (Fig. 1a, b, d, e). Continuous subcutaneous infusion of CNP attenuated the BLM-induced fibrotic changes (Fig. 1a–f). Quantitative histological analysis showed that BLM-induced fibrotic lesions were significantly smaller in mice treated with CNP than in those treated with vehicle alone (Fig. 1g). CNP significantly reduced the amount of hydroxyproline, an indicator of the amount of collagen, in BLM-administered lung (Fig. 1h). CNP treatment tended to improve body weight loss by BLM administration (Fig. 1i). BLM administration significantly augmented the number of inflammatory cells (total cells, macrophages, or neutrophils) in bronchoalveolar lavage (BAL) fluid (Fig. 1j–l). In BAL fluid, both total and individual cell counts were significantly lower in CNP-treated mice than in vehicle-treated mice. These results indicate that CNP can attenuate the fibrotic changes and the accumulation of inflammatory cells in BLM-induced pulmonary fibrosis.

### CNP attenuated the expression of cytokines induced by BLM in mouse lung

**Fig. 2:**
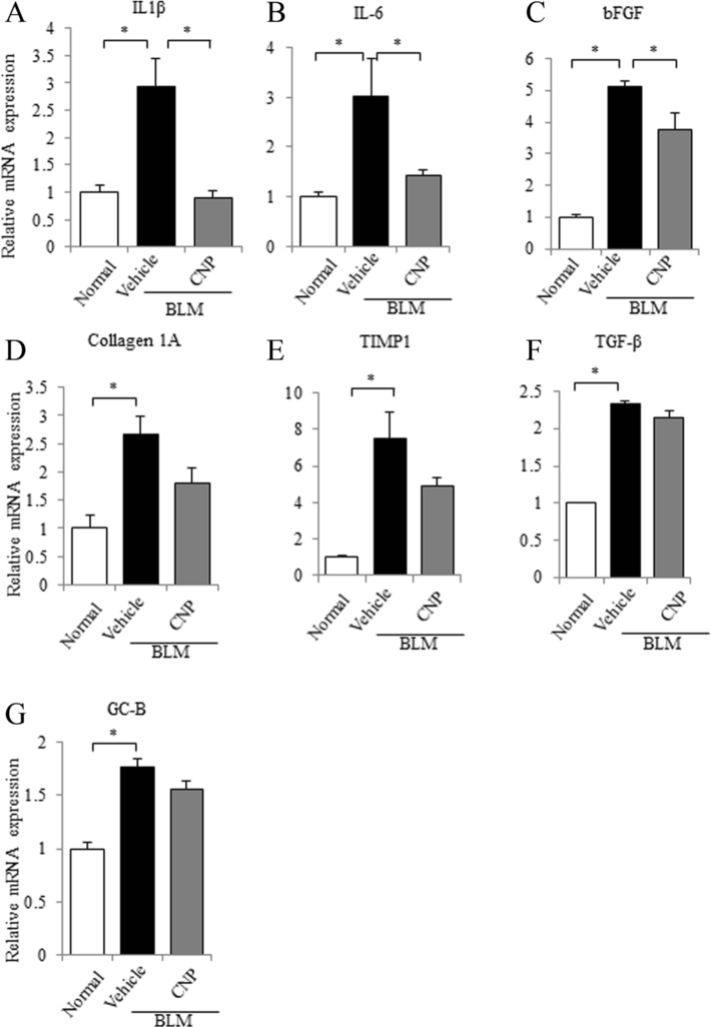
Continuous infusion of CNP attenuates the induction of pro-inflammatory and pro-fibrotic cytokines in BLM-administered mice. Quantitative RT-PCR analysis of interleukin (IL)-1β (**a**), IL-6 (**b**), basic fibroblast growth factor (bFGF) (**c**), collagen 1A (**d**), tissue inhibitor of metalloproteinase (TIMP) 1 (**e**), transforming growth factor (TGF)-β (**f**), and guanylyl cyclase (GC)-B (**g**) mRNA levels in lung tissues on day 14 after BLM administration was performed. The relative mRNA expression levels of each cytokine in normal control, BLM plus vehicle-administered, or BLM plus CNP-administered mice are shown. Values represent means ± SEM (*n* = 4 mice per group). *P < 0.05

To evaluate the anti-inflammatory and anti-fibrotic effects of CNP in BLM-induced lung fibrosis, we analyzed mRNA expression changes of pro-inflammatory cytokines (IL-1β and IL-6), pro-fibrotic cytokines and proteins (bFGF, TGF-β, TIMP1, and collagen 1A), and GC-B in the lungs. The mRNA expression levels of these genes were all significantly elevated on day 14 after BLM administration. CNP treatment significantly reduced the mRNA expression levels of IL-1β, IL-6, and bFGF compared with those in the vehicle control (Fig. 2a–c); the mRNA expression levels of collagen 1A, TIMP1, and GC-B were not statistically different (Fig. 2d, e). The gene expression of TGF-β was similar between the CNP-treated and vehicle-treated mice (Fig. 2f). These results indicate that CNP has the potential to attenuate the production of pro-inflammatory and pro-fibrotic cytokines and the collagen accumulation in pulmonary fibrosis.

### Generation of periostin-CNP transgenic mice

**Fig. 3:**
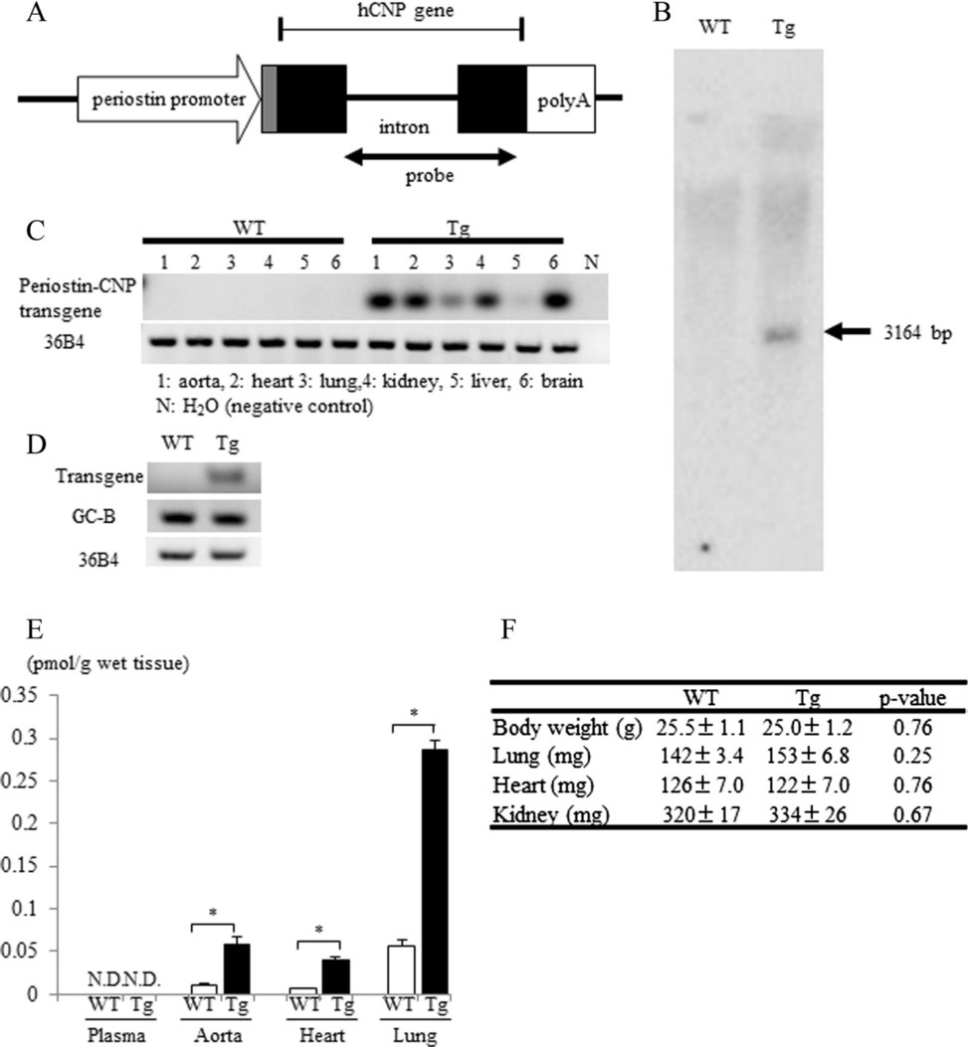
Generation of the periostin-C-type natriuretic peptide transgenic mouse (periostin-CNP Tg mouse). **a** Schematic representation of the periostin-CNP transgene. The transgene consisted of the periostin promoter (containing the 5′-untranslated region, shown as a gray box) linked to the human CNP gene (exons indicated by closed boxes) and an SV40 polyadenylation sequence. The probe (855 bp) used for Southern blot analysis is indicated by a double-headed arrow. **b** Southern blot analysis of EcoRV-digested mouse genomic DNA from a periostin-CNP Tg mouse and its wild-type (WT) littermate hybridized with the probe for hCNP transgene indicated in Fig. 3a. **c** The expression of the transgene was examined in aorta, heart, lung, kidney, liver, and brain by reverse transcribed–PCR. Tg, periostin-CNP transgenic mouse; WT, wild-type littermate. **d** The mRNA expression levels of the transgene and GC-B receptor were examined in primary cultured lung fibroblasts from a periostin-CNP Tg mouse and its WT littermate by PCR. A representative image of 3 independent experiments is shown. **e** The concentration of CNP in plasma, aorta, heart, and lung (WT, *n* = 3; Tg, *n* = 3). *P < 0.05. ND, not detected. **f** Body weight and weights of lung, heart and kidney. Values represent means ± SEM (WT, *n* = 7; Tg, *n* = 5)

To examine the anti-inflammatory and anti-fibrotic effects of CNP via fibroblasts in vivo, we generated a periostin-CNP transgenic (Tg) mouse model in which CNP was overexpressed (under the control of the periostin promoter) (Fig. 3a, b). We confirmed the transgene expression in various organs (aorta, heart, lung, kidney, liver, and brain) (Fig. 3c) and primary cultured lung fibroblasts from a periostin-CNP Tg mouse (Fig. 3d) by RT-PCR analysis. Expression of the transgene was observed in primary cultured lung fibroblasts from the periostin-CNP Tg mouse but not in those of its wild-type (WT) littermate; in contrast, expression of the GC-B gene was observed in primary cultured lung fibroblasts from both the periostin-CNP Tg mouse and its WT littermate (Fig. 3d). The concentration of CNP was significantly higher in organs (aorta, heart, and lung) of periostin-CNP Tg mice than those of WT littermates; in contrast, the CNP in plasma samples of both periostin-CNP Tg and WT mice were not detected (Fig. 3e). Body weight and organ size were not different between periostin-CNP Tg mice and WT littermates (Fig. 3f).

### BLM-induced pulmonary fibrosis was alleviated in periostin-CNP Tg mice

**Fig. 4:**
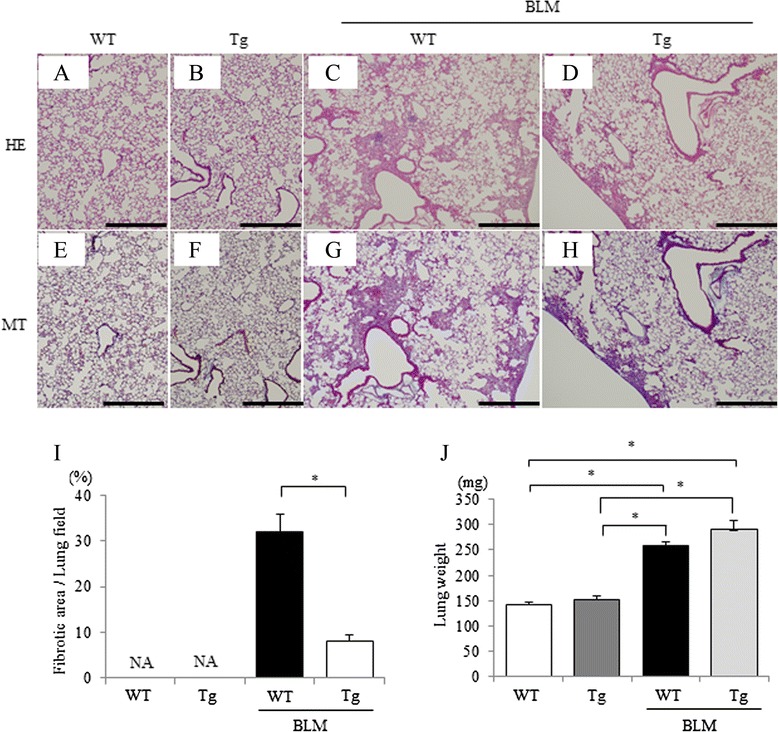
BLM-induced pulmonary fibrosis in periostin-CNP Tg mice. BLM (1 mg/kg) was administered intratracheally to periostin-CNP Tg mice and WT littermates on day 0, and the lung tissues were removed on day 14. Representative micrographs of lung tissue stained with hematoxylin-eosin (HE; upper panels) or Masson trichrome (MT; lower panels); WT littermates without BLM administration (**a**, **e**), periostin-CNP Tg mice without BLM administration (**b**, **f**), WT littermates with BLM administration (**c**, **g**), and periostin-CNP Tg mice with BLM administration (**d**, **h**) are shown. Scale bar: 500 μm. **i** Fibrotic area was analyzed by using image analyzing software and is expressed as a percentage of the whole lung field. Values represent means ± SEM (WT without BLM, *n* = 7; Tg without BLM, *n* = 7; WT with BLM, *n* = 8; Tg with BLM, *n* = 9). *P < 0.05. NA, not assessed because of no fibrotic area in normal lungs. **j** Lung weight. Values represent means ± SEM (WT without BLM, *n* = 7; Tg without BLM, *n* = 5; WT with BLM, *n* = 7; Tg with BLM, *n* = 5). *P < 0.05

We examined the in vivo anti-fibrotic effect of CNP via lung fibroblasts by using periostin-CNP Tg mice in the BLM-induced lung fibrosis model. There was no death in this experiment. In periostin-CNP Tg mice, BLM-induced pulmonary fibrotic change was markedly suppressed when compared with that in WT littermates (Fig. 4a–h). Quantitative histological analysis revealed that BLM-induced fibrotic lesions were significantly smaller in periostin-CNP Tg mice than in WT littermates (Fig. 4i), suggesting that CNP exerts its anti-fibrotic effect in the lung through fibroblasts. The lung weight was significantly increased by BLM administration (Fig. 4j).

### Expression of cytokines induced by BLM in mouse lung was alleviated in periostin-CNP Tg mice

**Fig. 5:**
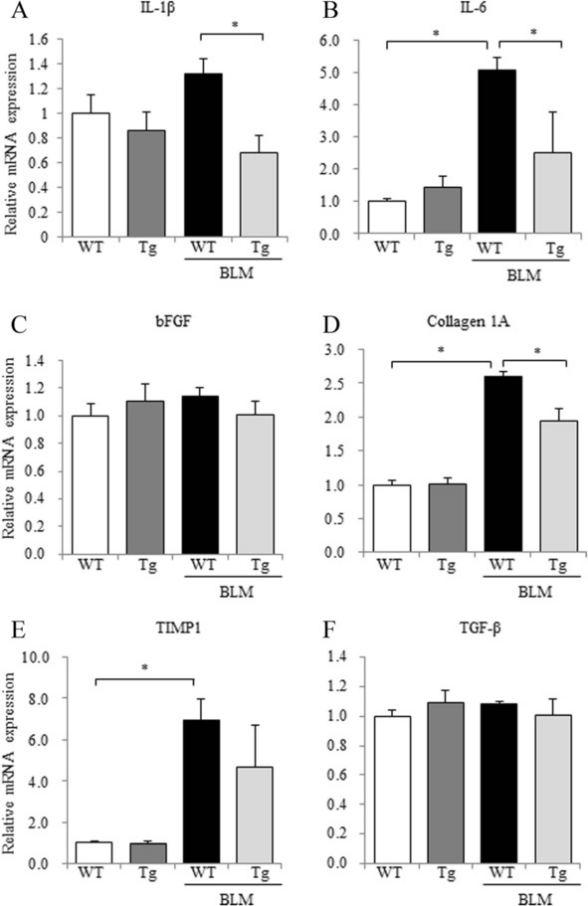
Changes in pulmonary inflammation and collagen deposition in periostin-CNP Tg mice after BLM administration. Quantitative RT-PCR analysis of IL-1β (**a**), IL-6 (**b**), bFGF (**c**), collagen 1A (**d**), TIMP1 (**e**), and TGF-β (**f**) in lung tissues at 14 days after BLM administration was performed. The relative mRNA expression levels (mean of WT littermates = 1) of each cytokine in WT littermates without BLM (*n* = 5), periostin-CNP Tg mice without BLM (*n* = 6), WT littermates with BLM (*n* = 9), or periostin-CNP Tg mice with BLM (*n* = 6) are shown. Values represent means ± SEM. *P < 0.05

To evaluate the anti-inflammatory and anti-fibrotic effects of CNP against BLM-induced lung fibrosis in periostin-CNP Tg mice, we analyzed the mRNA expression changes of IL-1β, IL-6, bFGF, TGF-β, TIMP1, and collagen 1A. In periostin-CNP Tg mice, the gene expression levels of IL-1β, IL-6, and collagen 1A were significantly decreased compared with those in WT littermates (Fig. 5a, b, d). TIMP1 mRNA expression was not significantly difference in periostin-CNP Tg mice and WT littermates (Fig. 5e). Neither TGF-β nor bFGF mRNA expression levels differed significantly between the groups (Fig. 5c, f). The results indicate that CNP produced anti-inflammatory and anti-fibrotic effects in the BLM-induced pulmonary fibrosis model in periostin-CNP Tg mice.

### Establishment of immortalized human lung fibroblasts stably expressing GC-B (LF^hTERT^/GC-B)

**Fig. 6:**
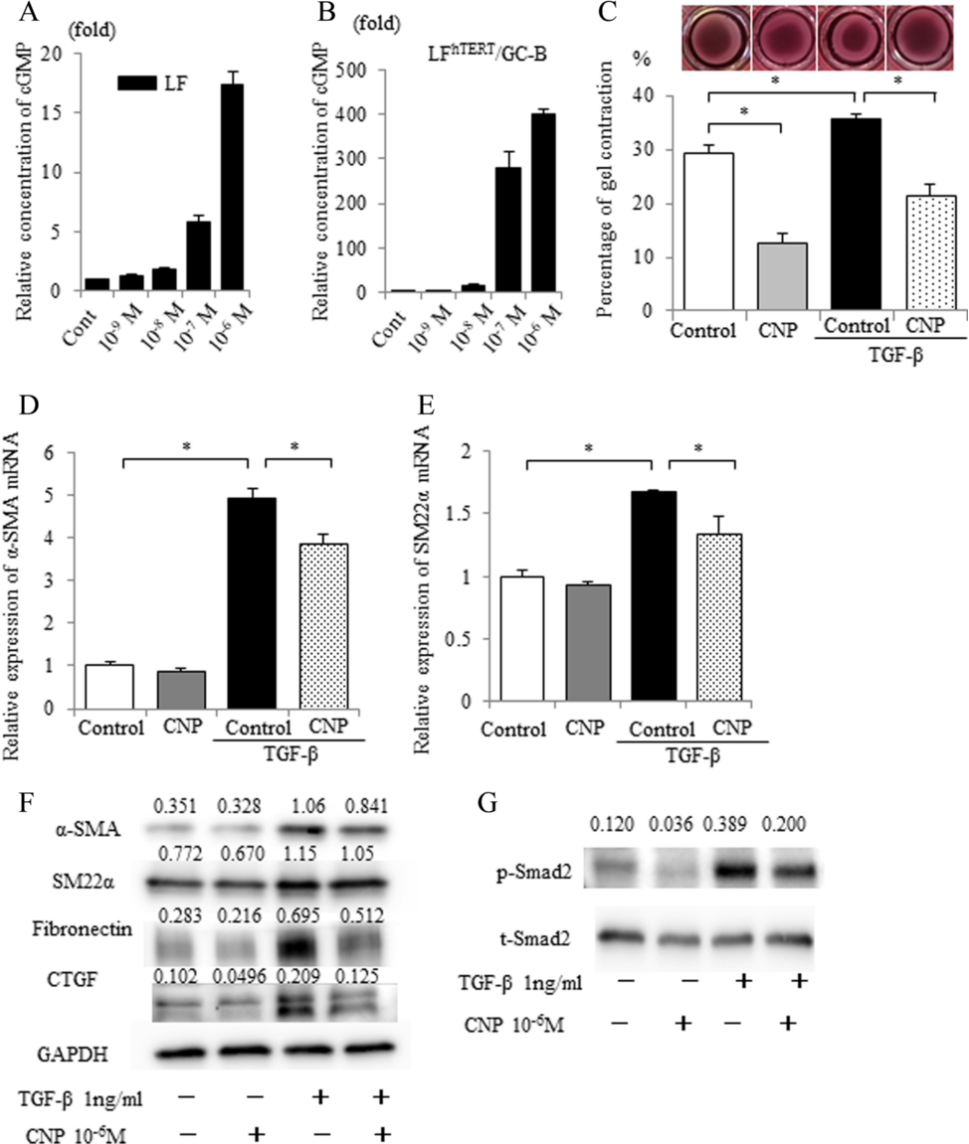
CNP attenuated TGF-β-induced fibroblast differentiation in human lung fibroblasts. Primary cultured human lung fibroblasts (LF) from a surgically resected specimen of a lung cancer patient (**a**) and its immortalized and GC-B stably expressed cell-line (LF^hTERT^/GC-B) (**b**) were treated with CNP at 10 nM to 1 μM in combination with 3-isobutyl-1-methylxanthine (0.5 mM). The cGMP concentrations in the cell lysate were measured 15 min after CNP-treatment. Relative concentrations of cGMP (mean of control without CNP = 1) are shown. Values represent means ± SEM. **c** Gels comprising type I collagen (0.75 mg/ml) and LF^hTERT^/GC-B cell suspension (5 × 10^5^ cells/ml) were maintained in serum-free DMEM with or without 1 ng/ml TGF-β. CNP was added to the medium at a final concentration of 1 μM every 24 h. After 3 days, the gels were released from the plate and their diameter was measured. The percentage of contraction is shown. Values represent means ± SEM. *P < 0.05. Results are representative of three independent experiments. **d** and **e** The relative mRNA expression of α-SMA (**d**) and SM22α (**e**). LF^hTERT^/GC-B cells were serum-starved for approximately 24 h. The cells were then pretreated with CNP (1 μM) in DMEM + 1 % FCS for 30 min and treated with or without TGF-β (1 ng/ml) for 24 h (for mRNA analysis) or 48 h (for protein analysis). Expression levels were normalized to that of 36B4 mRNA, and then relative expression levels were calculated (mean of control = 1). Values represent means ± SEM. *P < 0.05. Results are representative of three independent experiments. **f** Immunoblot analysis of α-smooth muscle actin (SMA), SM22α, fibronectin, connective tissue growth factor (CTGF), and glyceraldehyde 3-phosphate dehydrogenase (GAPDH). Cells were treated as in (**d**). Then cell lysates were prepared and subjected to immunoblot analysis. A representative blot of three independent experiments is shown. **g** The LF^hTERT^/GC-B cells were serum-starved for approximately 24 h. The cells were then pretreated with CNP (1 μM) in DMEM + 1 % FCS and treated with or without TGF-β (1 ng/ml) for 30 min. Cell lysates were immunoblotted for phospho-Smad 2 (p-Smad2) or total Smad 2 (t-Smad2). A representative blot of three independent experiments is shown with the ratio of the p-Smad2 to t-Smad2 signal intensities

Our observations that CNP exerted anti-inflammatory and anti-fibrotic effects in both the exogenous CNP-infusion and periostin-CNP Tg experimental models, without significant changes in TGF-β gene expression, prompted us to hypothesize that CNP suppresses downstream signals of TGF-β and differentiation of lung fibroblasts. To test this, we isolated lung fibroblasts (LFs) from a non-tumor region of lung from a lung cancer patient without ILD and confirmed that the fibroblasts produced cGMP following CNP treatment in a concentration-dependent manner (Fig. 6a). It is previously reported that the amount of GC-B receptor in cultured cells and cell lines decreased with the number of passages [38–40]. To facilitate in vitro studies of human LFs, LFs were immortalized and GC-B receptors were stably expressed to create the LF^hTERT^/GC-B cell line. We confirmed the CNP-induced dose-dependent elevation of cGMP in the LF^hTERT^/GC-B cell line (Fig. 6b).

### TGF-β–induced differentiation of human lung fibroblasts to myofibroblasts was attenuated by CNP

When stimulated with TGF-β, fibroblasts differentiate into myofibroblasts and show increased gel contractility in an extracellular matrix-remodeling assay [37]. Therefore we first examined whether stimulation of LF^hTERT^/GC-B cells with TGF-β also results in increased gel contractility. The ability of LF^hTERT^/GC-B cells to contract collagen gel was significantly increased by TGF-β stimulation, and this increase was reversed by CNP treatment (Fig. 6c). In response to TGF-β, LF^hTERT^/GC-B cells upregulated the production of α-SMA and SM22α, which was suppressed by CNP treatment (Fig. 6d, e). The protein expression levels of fibronectin and connective tissue growth factor (CTFG) were also upregulated by TGF-β stimulation and attenuated by CNP treatment (Fig. 6f). To elucidate the mechanism by which CNP inhibits TGF-β–mediated myofibroblast differentiation, we examined the influence of CNP on Smad phosphorylation, which occurs immediately following TGF-β stimulation [41]. As shown in Fig. 6g, CNP treatment suppressed TGF-β–induced phosphorylation of Smad2. These results suggest that CNP attenuates TGF-β–induced differentiation of fibroblasts into myofibroblasts via inhibition of the TGF-β/Smad2 signaling pathway.

### Human lung fibroblasts derived from patients with or without ILD express similar levels of GC-B to each other

**Fig. 7:**
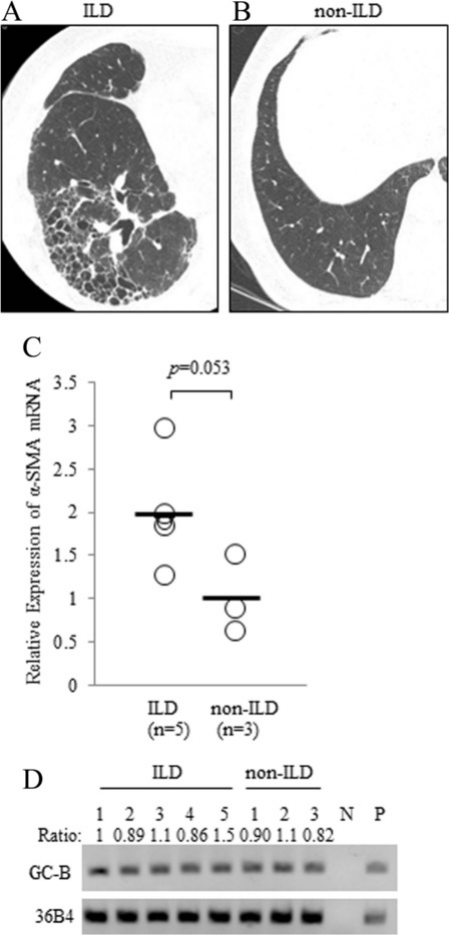
Human lung fibroblasts express GC-B to a similar extent with or without interstitial lung disease (ILD). Representative computed tomography images of human lung with (**a**) or without (**b**) ILD. **c** The expression of α-SMA was measured by qRT-PCR analysis in RNA extracts of primary cultured human lung fibroblasts that were obtained from surgically resected specimens of lung cancer patients with (*n* = 5) or without (*n* = 3) ILD. The α-SMA mRNA levels were normalized to 36B4 mRNA levels, and then relative expression levels (mean of normal = 1) were calculated. The bar represents the mean value of each group. **d** The mRNA expression of GC-B was examined in the primary cultured human lung fibroblasts by RT-PCR. N: negative control (water). P: positive control

To investigate whether CNP acts on human lung fibroblasts in a clinical setting, we obtained primary-cultured fibroblasts from the surgically resected lungs of lung cancer patients. Human distal parenchymal fibroblasts were isolated from the lung tissue of patients with interstitial lung disease (ILD; *n* = 5) or without interstitial lung disease (control; *n* = 3) (Fig. 7a, b). Demographic information for the patients who provided fibroblasts is summarized in Table 2. Fibroblasts used in the in vitro studies described above were obtained from patient number 1 without ILD. While the lung fibroblasts from ILD patients showed relatively high expression of α-SMA mRNA compared with those without ILD (Fig. 7c) as reported [7, 42], GC-B mRNA was expressed abundantly in both groups (Fig. 7d). These results suggest that CNP potentially affects human lung fibroblasts equally in patients with or without ILD.

**Table 2:** Demographic information for patients who provided fibroblasts

	Patient	Age/Sex	Histology	KL-6^a^ (U/mL)	SP-D^b^ (ng/mL)	FVC (L)	FEV1.0 (L)	TLC (L)	DLco (mL/min/mmHg)
With ILD	1	84/M	Sq	613	159	2.13	1.76	3.84	6.06
	2	69/M	Sq	622	159	3.07	2.23	4.29	8.79
	3	64/M	Ad	1129	321	2.77	2.27	4.25	12.0
	4	79/M	Sq	599	62.4	2.85	2.14	4.36	9.75
	5	77 M	Sq	658	103	2.71	2.21	4.32	12.9
Without ILD	1	81/F	Ad	ND^c^	ND^c^	1.95	1.45	3.24	9.84
	2	79/F	Sq	ND^c^	ND^c^	2.27	1.76	3.98	12.8
	3	81/M	Ad	ND^c^	ND^c^	3.17	2.18	5.39	16.3

*Definition of abbreviations*:

*ILD* interstitial lung disease, *Sq* squamous cell carcinoma, *Ad* adenocarcinoma, *SP-D* surfactant protein-D, *FVC* forced vital capacity, *FEV1.0* forced expiratory volume 1.0 (sec), *TLC* total lung capacity, *Dlco* diffusing capacity of the lung carbon monoxide

^a^Normal range of serum KL-6 is <500U/mL

^b^Normal range of serum SP-D is <110 ng/mL

^c^The serum levels of KL-6 and SP-D in the patients without ILD were not examined

## Discussion

Here, by using a bleomycin-induced lung fibrosis mouse model, we demonstrate that CNP has an anti-fibrotic effect in lungs through pulmonary fibroblasts. We show that BLM-induced pulmonary fibrosis is attenuated in both CNP-treated mice and periostin-CNP Tg mice by the CTP downregulating pro-inflammatory and pro-fibrotic cytokines and inhibiting collagen deposition. We reveal that CNP affects human lung fibroblasts in vitro and attenuates their activation by TGF-β via TGF-β-Smad2 signaling. These findings highlighted one of the mechanisms of anti-fibrotic effect of CNP that was unclear in the previous study [23]. In addition, we demonstrate that human lung fibroblasts express the GC-B receptor, regardless of the presence or absence of ILD in the patient of origin, suggesting that CNP could potentially have a therapeutic effect in humans.

Anti-inflammatory and anti-fibrotic effects of CNP have been reported in various tissues [16, 18–22]. Previous study showed that CNP is expressed in all major cell types in the lung during development and exerts effects on the airway epithelium [43, 44]. In lungs, CNP infusion inhibits the infiltration of inflammatory cells, including macrophages and monocytes, neutrophils, and lymphocytes in a monocrotaline-induced pulmonary hypertension rat model [45] and an LPS-induced acute lung injury mouse model [24]. These studies focused mainly on vascular endothelial cells as a site where CNP functions. CNP has been reported to inhibit the LPS-induced inflammatory reaction and expression of adhesion molecules in human endothelial cells in vitro [46]. Murakami et al. [23] reported that CNP modulated the BLM-induced inflammatory reaction by reducing the production of inflammatory cell-attracting chemokines. In our results, the vasoprotective effect of CNP could result partially in the attenuation of inflammatory cell infiltration induced by BLM via reduction of adhesion molecules. Although mRNA expressions of Col1A and TIMP-1 were not statistically different between vehicle-treated mice and CNP-treated mice, we speculate that the CNP-induced tendency of decrease in these mRNA expressions lead to the significant decrease in accumulation of hydroxyproline by reducing both production and decomposition of collagen. Inflammation is the initial step of fibrosis, and once fibrotic change occurs, fibroblasts and myofibroblasts increase in number due to proliferation of these cells [47]. Fibroblasts and myofibroblasts regulate chronic persistent inflammation and are the effector cells in the development of tissue fibrosis [4]. Since CNP has an anti-fibrotic effect in various tissues and regulates activated cardiac fibroblasts, we focused on the pulmonary fibroblasts as another target of CNP in the BLM-induced pulmonary fibrosis model.

Periostin, which is mainly produced by fibroblasts, is a recently characterized matricellular protein that binds to matrix proteins (collagen I, fibronectin, and tenascin-C) and interacts with several integrin molecules (i.e., α_v_, β_1_, β_3_, and β_5_) on cell surfaces, providing signals for tissue development and remodeling [28, 48]. Periostin is integral to wound healing, skin sclerosis, and fibrosis in myocardial infarction [49–51]. In the lung, periostin regulates inflammation and promotes extracellular matrix deposition in BLM-induced lung injury in mouse [28, 52], and also influences pathogenesis in human IPF [52]. Here, we generated mice overexpressing CNP by using the mice periostin promoter to focus on the effect of CNP in lung fibroblasts. Because all the examined organs (aorta, heart, lung, kidney, liver, and brain) contain fibroblasts in nature and periostin is dominantly but not specifically expressed by fibroblasts, expression of the transgene was observed in each organ of periostin-CNP Tg mice. We confirmed that the mRNA expression of the transgene was observed only in the cultured lung fibroblasts from periostin-CNP Tg mice (and not in those from WT littermates) and that the mRNA of GC-B receptor was expressed in both Tg and WT mice. Additionally, we confirmed that the concentration of CNP in the lung of periostin-CNP Tg mice was higher than the concentrations found in the other organs and in WT mice. These results demonstrate that periostin-CNP Tg is a model for elucidation of the anti-inflammatory and anti-fibrotic effects of CNP, which is expressed in part by fibroblasts and affects fibroblasts. CBP also affects fibroblasts that are found in lung tissue. Our results from studying BLM-induced lung fibrosis in these mice indicate that CNP affects not only endothelial cells but also fibroblasts and has the potential to attenuate the production of inflammatory cytokines and collagen accumulation in vivo. Because the concentration of CNP in plasma from both periostin-CNP Tg and WT mice was too low to be detected, the effects of CNP in this model was thought to be autocrine or paracrine rather than endocrine.

TGF-β, one of the most potent profibrotic cytokines, induces differentiation of fibroblasts to myofibroblasts [53]. As previously reported [8, 54], TGF-β is up-regulated in IPF, which leads to myofibroblast differentiation and enhanced secretion of extracellular matrix that, in turn, leads to the development of fibrotic foci [8, 55]. However, our data showed that CNP did not attenuate the mRNA expression of TGF-β in lungs in either the CNP administration or periostin-CNP Tg mouse model, while CNP attenuated the fibrotic changes in BLM-injured lungs. TGF-β is stored in extracellular matrix as a latent complex and becomes active through contractile forces generated by fibroblasts and myofibroblasts [56]. Fibroblasts and myofibroblasts express a similar level of total TGF-β (i.e., latent plus active TGF-β) to each other [56]. Our in vitro data showed that CNP treatment suppressed the TGF-β–induced increase of gel contractility and expression of myofibroblastic markers (α-SMA, SM22α, fibronectin, and CTGF) in human lung fibroblasts. Taken together, these findings suggest that the in vivo anti-fibrotic effect of CNP might be exerted via lung fibroblasts by disturbing the TGF-β–mediated transformation of lung fibroblasts to myofibroblasts.

Activated TGF-β ligands bind to a heteromeric complex of type I and type II receptors that transduce intracellular signals via phosphorylation of receptor-associated Smad2 and Smad3 [42, 57]. Phosphorylation of Smad2 and Smad3 followed by their nuclear translocation are critical steps in TGF-β signaling [42, 57]. B-type natriuretic peptide can prevent TGF-β–induced myofibroblast formation from cardiac fibroblasts [58]. Li et al. [59] reported that ANP/cGMP/PKG signaling intercepts the TGF-β signaling cascade in cardiac fibroblasts. Here, we revealed that CNP inhibited TGF-β–induced myofibroblast differentiation in human lung fibroblasts via the inhibitory effect of CNP on TGF-β-stimulated phosphorylation of Smad2. Considering that the bioactivity of CNP is mainly mediated by its specific receptor GC-B [10], CNP/GC-B/cGMP signaling may intercept the TGF-β signaling cascade in lung fibroblasts. Our in vitro study showed that CNP modulated at baseline without TGF-β stimulation. We speculate that this is because CNP suppressed not only TGF-β/Smad2 signal [40, 60]. The effect seemed to be mild at best by PCR. However, we think that the results of PCR and western blot analysis were not inconsistent because we assessed mRNA expression 24 h after stimulation and accumulated protein production 72 h after stimulation. Furthermore, we confirmed that GC-B is expressed in the human lung fibroblasts of patients with or without ILD. This suggests a therapeutic potential of CNP for patients with ILD including IPF. However, further study to clarify the detailed mechanism of action and appropriate dose and method of administration are warranted before clinical application. We have reported the effects of ANP in acute lung injury [61] and lung cancer metastasis [62] and we think that both CNP and ANP have anti-inflammatory effects in the lungs. We speculate that combined therapy of CNP and ANP may be more effective because their transmembrane receptors are different.

We have some limitations in this study. First, periostin is dominantly but not specifically expressed by fibroblasts and we should have selected the promoter which was more specific to fibroblast. However, we had difficulty in generating periostin-CNP Tg mice and it may be more difficult to generate CNP-Tg mice using the other promoter more specific to fibroblast. Second, the fact that GC-B mRNA is expressed in human lung fibroblasts does not provide any evidence that the protein is present and that downstream pathways are functional. Further study to disclose whether there is any difference in protein expression levels and downstream pathways of GC-B between ILD and non-ILD patients is warrant. We are performing a study on the assessment of plasma concentrations of CNP in humans, and then will plan the clinical trial to elucidate the effect of CNP in humans.

## Conclusions

Our results suggest that CNP exerts anti-inflammatory and anti-fibrotic effects in a BLM-induced lung fibrosis mice model through lung fibroblasts possibly by disturbing the TGF-β-Smad signaling pathway, thereby attenuating the transformation of the lung fibroblasts to myofibroblasts.
