# Erratum to: C-type natriuretic peptide ameliorates pulmonary fibrosis by acting on lung fibroblasts in mice

**DOI:** 10.1186/s12931-016-0429-1

**Published:** 2016-09-15

**Authors:** Toru Kimura, Takashi Nojiri, Jun Hino, Hiroshi Hosoda, Koichi Miura, Yasushi Shintani, Masayoshi Inoue, Masahiro Zenitani, Hiroyuki Takabatake, Mikiya Miyazato, Meinoshin Okumura, Kenji Kangawa

**Affiliations:** 1 Department of Biochemistry, National Cerebral and Cardiovascular Center Research Institute, 5-7-1, Fujishirodai, Suita-city, Osaka 565-8565 Japan; 2 Department of General Thoracic Surgery, Osaka University Graduate School of Medicine, Suita-City, Osaka Japan; 3 Department of Regenerative Medicine and Tissue Engineering, National Cerebral and Cardiovascular Center, Suita-City, Osaka Japan; 4 Department of General Thoracic Surgery, Kyoto Prefectural University of Medicine, Kyoto-City, Kyoto Japan

## Erratum

In the original publication of this article [1], the acknowledgements and contributions sections were incorrectly listed.

These should have been:

**Author’s contributions**

Conception and Design: TK, TN, KK. Analysis and Interpretation: TK, TN, JH, HH, KM. Collecting the data: TK, JH, HH, KM, YS, MZ, HT. Drafting the manuscript for important intellectual content: TK, TN, JH, HH, KM, YS, MI. Obtaining the funding: TK, TN, MM, OM, KK. All authors have approved the version of the submitted manuscript.

**Acknowledgements**

We thank R. Weingerg for providing the hTERT immortalizing gene. We thank K. Sasai and T. Akagi for providing the lentivirus vector expressing hTERT. This work was supported in part by Grant-in-Aid for Young Scientists B (26861135) and the Intramural Research Fund for Cardiovascular Diseases of the National Cerebral and Cardiovascular Center to T. Kimura, Japan Research Foundation for Clinical Pharmacology, Kobayashi Foundation for Cancer Research, Mochida Memorial Foundation for Medical and Pharmaceutical Research, Uehara Memorial Foundation, and Takeda Science Foundation to T. Nojiri, Grant‐in‐Aid for Scientific Research on Innovative Areas (Research in a proposed research area) from the Ministry of Education, Culture, Sports, Science and Technology of Japan, and Japan Vascular Disease Research Foundation to K. Kangawa.
